# Microbial Community Structure and Diversity in an Integrated System of Anaerobic-Aerobic Reactors and a Constructed Wetland for the Treatment of Tannery Wastewater in Modjo, Ethiopia

**DOI:** 10.1371/journal.pone.0115576

**Published:** 2014-12-26

**Authors:** Adey Feleke Desta, Fassil Assefa, Seyoum Leta, Francesca Stomeo, Mark Wamalwa, Moses Njahira, Djikeng Appolinaire

**Affiliations:** 1 Institute of Biotechnology, Addis Ababa University, Addis Ababa, Ethiopia; 2 Centre for Environmental Science, College of Natural Sciences, Addis Ababa University, Addis Ababa, Ethiopia; 3 Biosciences eastern and central Africa- International Livestock Research Institute (BecA-ILRI) Hub, Nairobi, Kenya; Purdue University, United States of America

## Abstract

A culture-independent approach was used to elucidate the microbial diversity and structure in the anaerobic-aerobic reactors integrated with a constructed wetland for the treatment of tannery wastewater in Modjo town, Ethiopia. The system has been running with removal efficiencies ranging from 94%–96% for COD, 91%–100% for SO4^2-^ and S^2-^, 92%–94% for BOD, 56%–82% for total Nitrogen and 2%–90% for NH_3_-N. 16S rRNA gene clone libraries were constructed and microbial community assemblies were determined by analysis of a total of 801 unique clone sequences from all the sites. Operational Taxonomic Unit (OTU) - based analysis of the sequences revealed highly diverse communities in each of the reactors and the constructed wetland. A total of 32 phylotypes were identified with the dominant members affiliated to Clostridia (33%), Betaproteobacteria (10%), Bacteroidia (10%), Deltaproteobacteria (9%) and Gammaproteobacteria (6%). Sequences affiliated to the class Clostridia were the most abundant across all sites. The 801 sequences were assigned to 255 OTUs, of which 3 OTUs were shared among the clone libraries from all sites. The shared OTUs comprised 80 sequences belonging to Clostridiales Family XIII Incertae Sedis, Bacteroidetes and unclassified bacterial group. Significantly different communities were harbored by the anaerobic, aerobic and rhizosphere sites of the constructed wetland. Numerous representative genera of the dominant bacterial classes obtained from the different sample sites of the integrated system have been implicated in the removal of various carbon- containing pollutants of natural and synthetic origins. To our knowledge, this is the first report of microbial community structure in tannery wastewater treatment plant from Ethiopia.

## Introduction

The leather industry is the second largest economic sector that contributes to foreign exchange earnings in Ethiopia [1]. Currently, 26 tanning industries are present in the country producing semi-finished and finished hides and skins [2]. Despite its benefit, the leather industry is characterized by the generation of a large amount of liquid waste constituting pollutants such as organic and inorganic matter, total dissolved solids as well as a variety of synthetic compounds [3], [4]. Due to the complex nature and excessive levels of the pollutants, treatment of tannery wastewater has become an important issue for pollution control in leather producing countries [5], [6]. Untreated tannery effluents can cause severe environmental pollution affecting surface and underground water resources [7], [4], human habitats and living systems [6].

Very few of the existing tanning industries in Ethiopia have treatment plants that enables them meet the requirements for effluent quality standards [8], [9]. As a result, surface and underground water pollution has become a major problem that needs to be addressed by establishing cost- effective wastewater treatment options.

Various physico-chemical [10], [11], oxidation- based [12], [13] and biological (including phytoremediation) (reviewed in [14]) systems have been used for the treatment of wastewater from tanning industries. More emphasis has been given to the physico- chemical and oxidation systems than to biological treatment methods owing to the high BOD and complex nature of the tannery wastewater [15], [16]. The employment of these methods, however, is costly because of their chemical and energy demanding characteristics [17], [18].

Integrated biological treatment methods have been practised by coupling or integrating two or more biological processes for the treatment of mainly municipal and rarely industrial wastewaters. A study showed a cost- effective and efficient treatment of municipal wastewater involving pond systems integrated with constructed wetland system [19]. A recent bench-scale study which coupled intermittent high pressure sequential bioreactor with sand filtration system has proven the high efficiency of the system in pollutants removal and energy consumption [20]. Although integrated biological systems have been characterized by their low operating costs and efficiency in pollutant removal [21], [22], they are not widely applied for the treatment of highly polluted industrial effluents such as tannery wastewater.

Besides the management of conventional process parameters, stable performance of any biological wastewater treatment system can be achieved by understanding and manipulating the microbial communities residing in the system [23]. Investigation of microorganisms responsible for efficient reduction of pollutants in various biological wastewater treatment plants have been conducted for many years [24], [25], [26]. Microbial studies in tannery wastewater treatment plants have been successfully conducted with regard to detection, isolation and characterization of bacteria involved in different natural recycling processes such as sulfur oxidation (27), denitrification [28] and phenol degradation [29] in a microcosm.

Apart from the conventional methods of harvesting and characterizing of microorganisms, the use of culture-independent molecular techniques has revolutionized the identification of microbial communities from various natural habitats (Reviewed in [30]) and wastewater treatment sites [31]. Microbial diversity analyses of biological reactors employing culture- independent tools have revealed the complex microbial diversity and structure of these ecosystems [32], [33]. Lefebvre and colleagues [34] surveyed the diversity of microorganisms in four hypersaline wastewater treatment plants, three of which from tanneries using 16S rRNA gene clone library – based sequencing. They reported high microbial diversity constituting 14 different bacterial phylotypes. A recent DGGE fingerprinting-based microbial study on constructed wetland systems for tannery wastewater revealed the presence of diverse and distinct bacterial assemblages inhabiting the different macrophytes [35], [36]. However, data on microbial communities from integrated anaerobic- aerobic- constructed wetland treatment processes for the efficient treatment of tannery wastewater are lacking. The current study shows the composition, structure and diversity of the bacterial communities in an integrated anaerobic- aerobic- constructed wetland system treating tannery effluent in Modjo, Ethiopia.

## Materials and Methods

### Ethics statement

This biological treatment system is established in the vicinity of a privately owned tannery (37P 512542–512587mE and 951301–951400mN). Permission to use the land for the construction of the integrated biological treatment system was granted by the owner. The system was able to treat half the wastewater volume (150 m^3^) from the tannery. The tannery management team usually gets an update about the various research findings and recommendations for better performance. The study did not involve endangered or protected species.

### Description of study site and pilot- scale treatment system

The study was conducted on a pilot-scale biological wastewater treatment plant (WWTP) which was installed in the premises of a privately owned tannery in Modjo Town, Ethiopia, 70 km south of the capital Addis Ababa. The system consists of tanks for primary screening and grit removal, two anaerobic reactors each with volume of 25 m^3^ and having 3.6 m diameter and 3.3 m height; an aerobic reactor with a volume of 50 m^3^ having 4 m diameter and 3.3 m height, followed by a vertical- flow constructed wetland (31 m long, 16.40 m wide, 1.30 m deep and filled with medium- sized gravel ranging from 0.6 to 2 cm size) vegetated with the perennial grass *Phragmites australis* (Cav.). Wastewater was fed into the anaerobic reactors with hydraulic retention time of 24 hrs. The effluent from the anaerobic reactors was channelled to the aerobic reactor fitted with aeration pump and mixing for 12 hrs. The effluent from the aerobic reactor was fed into a collection tank from where it was continuously fed into the constructed wetland with a loading rate of 120 Kg/BOD_5_/ha/day. The pilot WWTP has been running since September 2010 and performance of the integrated system has been recorded as part of a routine monitoring of the system.

### Wastewater sampling and analytical methods

To evaluate the performance of the integrated system, samples of raw and treated wastewater were taken on the 6^th^, 13^th^ and 20^th^ of January 2012. The water samples were analyzed for selected parameters to assess the biological degradation of the pollutants. Chemical Oxygen Demands (COD), total N, NH_3_-N, NO_3_-N NO_2_-N, SO_4_^2-^ and S^2-^ were measured spectrophotometrically (DR/2012, HACH, Loveland, USA) according to the company's instructions. Biological Oxygen Demands (BOD), total suspended solids and volatile suspended solids were determined following standard methods [37]. Total chromium was determined using direct air- acetylene flame method of atomic absorption spectroscopy (AAS) according to standard methods [37]. Total suspended solids (TSS) were analysed using gravimetric method involving dry weight. Salinity and pH were measured using conductivity meter (ELEMETRON, CC401, Spain) and pH meter (Jenway Ltd., England).

### Sludge sampling

**Figure 1:**
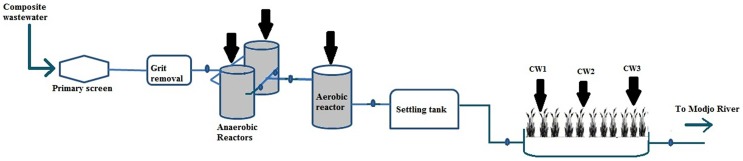
Schematic presentation of the pilot tannery effluent treatment site consisting of anaerobic-aerobic reactors integrated with constructed wetland system. Black arrows show sampling sites.

For analysis of microbial community composition in the treatment system, sludge samples were collected from the anaerobic and aerobic reactors. In order to achieve maximum recovery as well as representative information on microbial populations from the constructed wetland system, samples were collected from around the root zone of the plants in three randomly selected spots of the wetland (Fig. 1) and designated as CW1, CW2 and CW3. Sampling was done on the same dates of wastewater sampling. All samples were collected in duplicate in sterile containers at each sampling time and stored at −80°C until further analysis

### Genomic DNA extraction and PCR amplification

DNA was extracted from the duplicate sludge samples using ZR soil microbe DNA kit (Zymo Research, CA., USA) following instructions provided in the kit. Extracted DNA was visualized using 0.8% agarose gel pre-stained with GelRed (Biotium Inc) and run in 0.5X TBE buffer and electrophores was carried out at 100V for 20 minutes. DNA purity (A260/A280 and A260/A230) and quantification were measured with a NanoDrop ND-1000 spectrophotometer (Thermo Scientific).

Bacterial 16S rRNA gene fragments were amplified by PCR using the primers 27F (5′AGAGTTTGATCMTGGCTCAG 3′) and 1492R (5′ GGTTACCTTGTTACGACTT 3′) [38]. PCR was performed in 25 ul reaction mixtures containing 0.2 µl Dream Taq (Fermentas, Lithuania), 2.5 µl 10X Taq buffer, 0.5 µlof each deoxynucleoside triphosphate (10 mM), 1 µl (0.4 pmol) of each primer (Bioneer corporation, Korea), 18.8 µl sterile filtered MilliQ water and 1 µl of template DNA (30 ng/µl). To minimize PCR bias, DNA extracts from the duplicate samples were pooled and served as the template DNA. Amplification was performed using GeneAmp PCR System 9700 (Applied Biosystems), with the cycle parameters as follows: 4 min at 94°C and 30 cycles of 45 s at 94°C, 1 min at 58°C an 2 min at 72°C, followed by a 10-min extension step at 72°C [39]. The results were checked by electrophoresis on a 1.5% agarose gel.

### 16S rRNA gene clone library construction and sequence analysis

16S PCR products amplified from the DNA extracts were purified using a QIAquick kit for PCR purification (QIAGEN, Germany). For maximum coverage of the PCR products during clone library construction, the purified products collected from the each site at three different time intervals were pooled. Cloning was performed using InsTAclone PCR cloning kit (Fermentas, Lithuania). A total of five clone libraries, one for each site, were constructed. From each library, 200 to 250 colonies were picked and screened for the appropriate insert size by colony PCR using vector-specific primers M13 (-21) (5′- TGT AAA ACG ACG GCC AGT-3′) and M13 reverse (−29) (5′CAG GAA ACA GCT ATG ACC-3′) [38]. PCR products were purified with Ethanol – sodium acetate purification as described in Koch et al., [39]. Amplified Ribosomal DNA Restriction Analysis (ARDRA) using single digests by HhaI and MspI was performed to dereplicate and classify the clones into phylotypes based on their unique restriction pattern. Partial DNA sequencing of the M13 amplicons was performed using a BigDye Terminator V 3.1 Cycle Sequencing Kit (Applied Biosystems) and the forward primer 27F. Electrophoresis and data collection were carried out on an ABI 3730 Genetic Analyzer (Applied Biosystems). The resulting sequence chromatograms were edited using Sequencher version 5.0 (Gene Codes Corporation, MI, USA).

### Statistical analyses

Sequences were edited and trimmed using CLC main workbench version 6.6.2 (CLC Bio, Aarhus, Denmark). Sequence alignment, chimeras checking, distance calculation, clustering and selection of non- redundant sequences were performed using MOTHUR version 1.25.0 [40]. Sequence identification was performed using The Ribosomal Database Project (RDP) Classifier, a naive Bayesian classification method [41]. OTU assignment and search for shared OTUs, rarefaction analysis, Chao I and the Shannon-Wiener (H) and Simpson's (D) diversity indices were calculated using MOTHUR. For estimating community differences in the five sampling sites, sequences were analyzed by Unifrac analysis using MOTHUR. Clustering of the sample sites based on their microbial composition was performed using non- metric multi dimensional scaling (n-MDS) based on Bray- Curtis dissimilarity index using PAST (version 2.08). Data of the bacterial composition of each site was presented in a bar chart using SPSS (version 16.0). Representative sequences were deposited in Genbank under the accession numbers KC110157 to KC110593.

## Results

### Performance of the integrated biological tannery wastewater treatment plant

The untreated tannery wastewater channelled into the integrated system was characterized by its high concentration of BOD, COD and Sulphate ranging from 4551–5201 mg/l, 11180–13770 mg/l and 200–1600 mg/l, respectively. Total N, ammonia N and sulphide concentrations ranged from 125–258 mg/l, 8–490 mg/l and 45–62 mg/l, respectively. The concentrations of total suspended solids, total dissolved solids and volatile suspended solids ranged from 890–1460 mg/l, 8620–11780 mg/l and 27171–27680 mg/l respectively. Effluent from the chrome- tanning section with high levels of total chromium (an average of 27574.25 mg/l) was segregated in a separate channel and thus the total chromium concentration in the influent wastewater was small ranging from 16–41 mg/l. The high pH indicated the alkalinity of the wastewater (Table 1).

**Table 1:** Average characteristics of the influent and effluent wastewaters for the feeds at the time of sludge sampling (concentrations are in mg/l, except for pH).

Parameter	Influent	Effluent	% Removal
TN	245.25±76	62.75±14	74
SO_4_^2-^	800±505	35±61	96
TP	15.33±1	4.23±2	72
S^2-^	55.50±6	4.91±3	91
NO_3_-N	310±203	40.25±28	87
NO_2_-N	2.08±3	0.03	99
NH_3_-N	287.70±178	44.28±26	85
COD	12547.50±3910	395±139	97
BOD	4886.26±266	308.91±24	94
Salinity	9470.50±1335	2593.69±344	73
TSS	1155±203	92±11	92
VSS	27482.75±197	2272.75±724	92
Total Cr	27.25±3	0.95	97
pH	10.40±0.3	7.66±0.1	

TN, total Nitrogen; TP, total Phosphorous; TSS, total suspended solids; VSS, volatile suspended solids; Total Cr, Total chromium.

The overall performance of the integrated biological treatment system in the removal of these pollutants ranged between 70–99%. The effluent parameters obtained for the BOD, COD, sulphate (SO_4_^2-^), sulphide (S^2-^), nitrate-nitrogen (NO_3_-N) and ammonia –nitrogen (NH_3_-N) were in line with the provisional emission limit values set for tannery effluents in Ethiopia (EPA 2003). The removal efficiency for total nitrogen was low (74%) and its concentration in the effluent (62.75±14 mg/l) was slightly above the discharge limit (60 mg/l). Likewise, the concentration of the effluent BOD was higher than the standard limit of discharge though the removal efficiency was above 80%.

**Figure 2:**
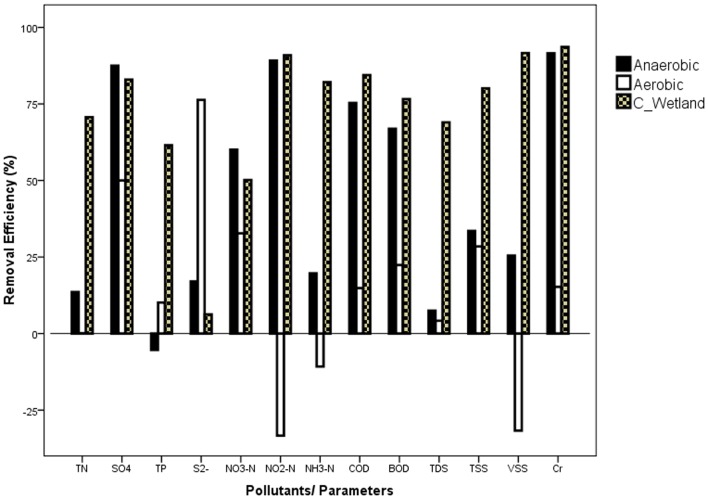
Comparative performances of the anaerobic and aerobic reactors; and the constructed wetland system.

When considering the relative treatment efficiency of the individual components that make up the system, the constructed wetland performed with the highest removal efficiencies of total nitrogen, total phosphorus, nitrite nitrogen, and ammonia nitrogen, COD, BOD, salinity, TSS and VSS (ranging from 70–93% removal). The anaerobic system performed well in the removal of sulphate, nitrate nitrogen, nitrite nitrogen and chromium, ranging from 60–91%. The aerobic system contributed to the removal of reduced sulphur (S^2-^), accounting for 76% although its contribution to the removal of total nitrogen was only less than 1% (Fig. 2).

### Microbial composition in the sludge and rhizosphere

A total of 1091 clones were screened by partial sequencing 16S rDNA inserts and 801 sequences were found to belong to 31 phyla. 761 sequences were unique and were clustered within 255 operational taxonomic units (OTUs) on the basis of at least 97% sequence similarity. Close to 27% of the original clone sequences were removed based on the criteria for screening sequences in MOTHUR. No phylotypes were inferred from the ARDRA analysis using either HhaI or MspI, as almost every clone exhibited unique band pattern. The phylum most represented in all the systems was that of Firmicutes, which contained 40% of all the sequences analysed, followed by Proteobacteria and Bacteroidetes, representing 30% and 15% of the sequences, respectively. Sequences of the phyla Synergistetes and Acidobacteria accounted for 2.8% and 1% respectively, while sequences that belonged to unclassified bacteria accounted for 3.6% of the total sequences. The phyla Actinobacteria, Chloroflexi, Tenericutes, Spirochaetes, Planctomycetes, Verrucomicromicrobia, Nitrospira and the uncultured candidate division SR1 each represented by less than 1% of the total sequences, comprised a total of 3.5% of the sequences.

**Figure 3:**
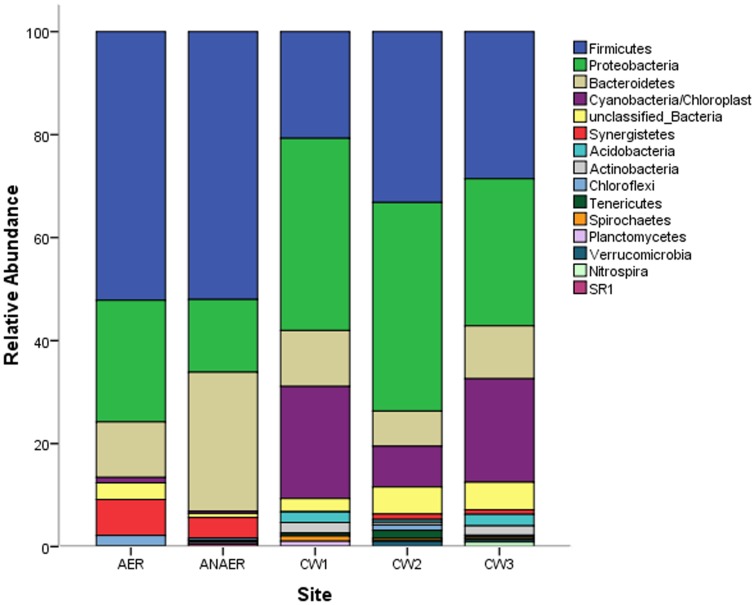
Composition and abundance of observed bacterial phyla based on the 16S rRNA clone libraries constructed from anaerobic, aerobic reactors and three constructed wetland sites arranged in a series.

Considering each of the treatment components, the phylum Firmicutes was represented by 53% of the sequences in the aerobic, 52% in the anaerobic, 44%, 43% and 31% in the three constructive wetland sites CW1, CW2 and CW3, respectively (Fig. 3). Proteobacteria were represented by 24% of the sequences in the aerobic reactor, 14% in the anaerobic reactor, 44%, 43% and 31% in CW1, CW2 and CW3 sites, respectively. The third largest phylum, Bacteroidetes was represented by 11% of the sequences in the aerobic reactor, 27% in the anaerobic reactor, 13%, 7% and 11% in CW1, CW2 and CW3 sites, respectively. Sequences of the phylum Cyanobacteria were retrieved only from the constructed wetland sites and accounted for 7% in CW1, 3% in CW2 and 12% in CW3 (Fig. 3).

**Figure 4:**
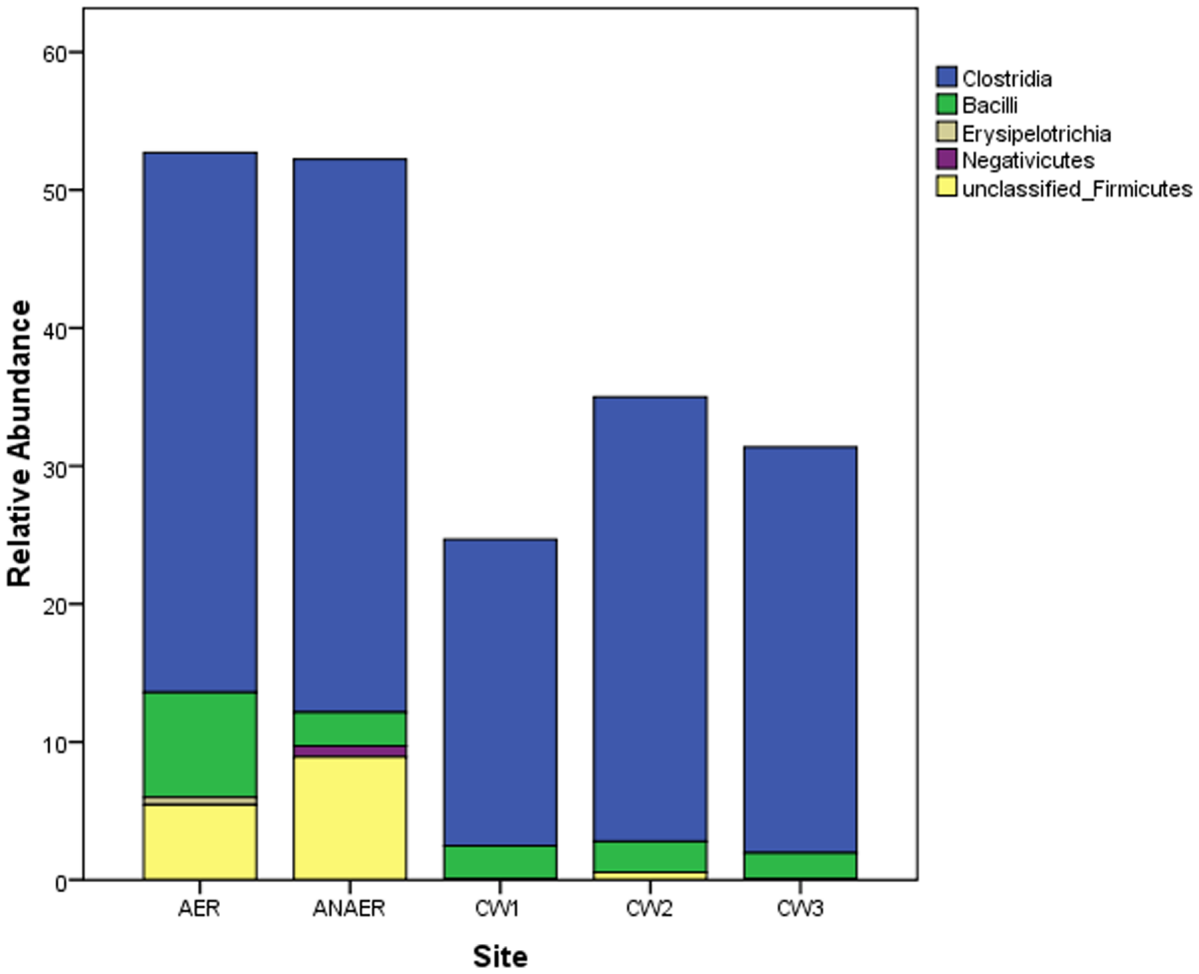
Class- level distributions of sequences affiliated to the dominant phylum Firmicutes based on the 16S rRNA gene clone libraries from anaerobic- aerobic reactors and three constructed wetland sites.

Among the Firmicutes, Clostridia was the most abundant class representing about 40% of the sequences in the aerobic and anaerobic reactors and 22% 32% and 29% in CW1, CW2, CW3, respectively. Class Negativicutes was retrieved only from the anaerobic site representing only 1% of the sequences. On the other hand, the class Erysipelotrichia was retrieved only from the aerobic site, representing 1% of the total sequence in the site. Unclassified Firmicutes comprised 5% of the aerobic, 9% of the anaerobic and 1% of the CW2 site (Fig. 4).

Some of the most abundant sequences belonging to Clostridia were closely related to the genera *Proteiniclasticum, Tissierella* and *Anaerovorax*, which belong to the families Clostridiaceae 1, Clostridiales Incertae Sedis XI and XIII, respectively (Table 2). Sequences belonging to the genera *Proteocatella* and *Tissierella* were localized in the anaerobic and aerobic reactors, and were not detected in the constructed wetland, while sequences similar to the genus *Acidaminobacter* were localized only in the constructed wetland (Table 2).

**Table 2:** Genus- level distribution of the different families of the dominant class Clostridia in the different components of the integrated reactor.

	Relative abundance (%)					
Genus	Aero	Anaero	CW1	CW2	CW3	Family
Tissierella	20	14	0	7	2	Clostridiales_IncertaeSedis XI
Proteiniclasticum	16	18	50	22	46	Clostridiaceae 1
Trichococcus	9	2	0	0	0	Carnobacteraceae
Anaerovorax	8	12	17	19	12	Clostridiales_IncertaeSedis XIII
Proteocatella	7	5	0	2	0	Peptostreptococcaceae
Acetivibrio	5	2	8	2	0	Ruminococcaceae
Alkalibacillus	5	1	0	0	0	Bacillaceae II
Saccharofermentans	3	5	0	2	7	Ruminococcaceae
Acetonema	3	0	0	0	0	Veillonellaceae
Anaerosinus	3	0	0	0	0	Veillonellaceae
Cellulosilyticum	2	0	0	2	0	Lachnospiraceae
Schwartzia	2	14	0	0	0	Veillonellaceae
Acetoanaerobium	2	6	0	2	0	Peptostreptococcaceae
Alkalibaculum	2	2	0	0	0	Eubacteriaceae
Pelospora	2	1	0	0	2	Syntrophomonadaceae
Alkalibacter	2	0	0	0	0	Eubacteriaceae
Thermodesulfobium	2	0	0	0	0	Thermodesulfobiaceae
Sedimentibacter	1	1	0	0	0	Clostridiales_IncertaeSedis XI
Desulfitibacter	1	0	0	4	5	Peptococcaceae 1
Eubacterium	1	0	0	4	0	Eubacteriaceae
Fusibacter	1	0	0	2	0	Clostridiales_IncertaeSedis XII
Papillibacter	1	0	0	2	0	Ruminococcaceae
Succinispira	1	0	0	2	0	Veillonellaceae
Flavonifractor	1	0	0	0	2	Ruminococcaceae
Anaerotruncus	1	0	0	0	0	Ruminococcaceae
Clostridium XII	1	0	0	0	0	IncertaeSedis XI
Clostridium XVIII	1	0	0	0	0	Erysipelotrichaceae
Dendrosporobacter	0	2	4	0	0	Veillonellaceae
Sporanaerobacter	0	2	0	0	0	Clostridiales_IncertaeSedis XI
Enterococcus	0	2	8	0	0	Enterococcaceae
Gracilibacter	0	2	0	2	2	Gracilibacteraceae
Oscillibacter	0	2	0	0	0	Ruminococcaceae
Phascolarctobacterium	0	2	0	0	0	Veillonellaceae
Acetobacterium	0	1	0	0	0	Eubacteriaceae
Butyricicoccus	0	1	0	0	0	Ruminococcaceae
Caldicoprobacter	0	1	0	0	0	IncertaeSedis IV
Desulfonispora	0	1	0	0	0	Peptococcaceae 1
Hydrogenoanaerobacterium	0	1	0	0	0	Ruminococcaceae
Lutispora	0	1	0	0	0	Gracilibacteraceae
Soehngenia	0	1	0	0	0	Clostridiales_IncertaeSedis XI
Ethanoligenens	0	0	4	4	2	Ruminococcaceae
Desulfitispora	0	0	4	0	2	Peptococcaceae 1
Clostridium sensu strict	0	0	4	0	0	Clostridiaceae 1
Acidaminobacter	0	0	0	4	2	Clostridiales_Incertae Sedis XII
Dethiobacter	0	0	0	4	0	Natranaerobiaceae
Exiguobacterium	0	0	0	2	2	Bacillales Incertae Sedis XII
Clostridium XI	0	0	0	2	0	Peptostreptococcaceae
Clostridium XlVa	0	0	0	2	0	Lachnospiraceae
Dehalobacter	0	0	0	2	0	Peptococcaceae 1
Dethiosulfatibacter	0	0	0	2	0	IncertaeSedis XI
Finegoldia	0	0	0	2	0	Clostridiales_IncertaeSedis XI
Thermacetogenium	0	0	0	2	0	Thermoanaerobacteraceae
Anaerofustis	0	0	0	0	2	Eubacteriaceae
Pseudoflavonifractor	0	0	0	0	2	Ruminococcaceae
Syntrophothermus	0	0	0	0	2	Syntrophomonadaceae
Thermobrachium	0	0	0	0	2	Clostridiaceae 1

**Figure 5:**
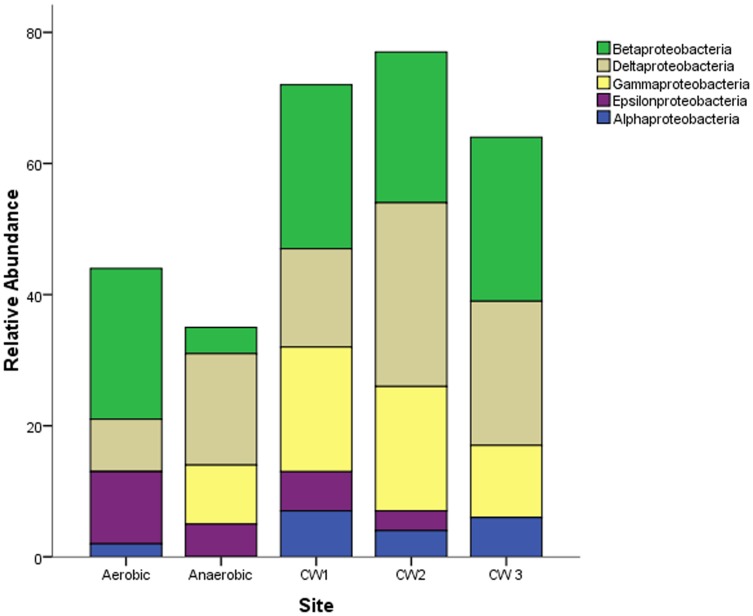
Class/Division - level distribution of sequences affiliated to the phylum Proteobacteria based on the 16S rRNA gene clone libraries from anaerobic- aerobic reactors and three constructed wetland sites.

Class/division- level distribution of sequences belonging to the phylum Proteobacteria showed that class Betaproteobacteria were the second most dominant sequences in the aerobic reactor (13% of the total sequences) and in the constructed wetland sites (15, 13 and 12% of the sequences in CW1, CW2 and CW3, respectively) (Fig. 5).

**Figure 6:**
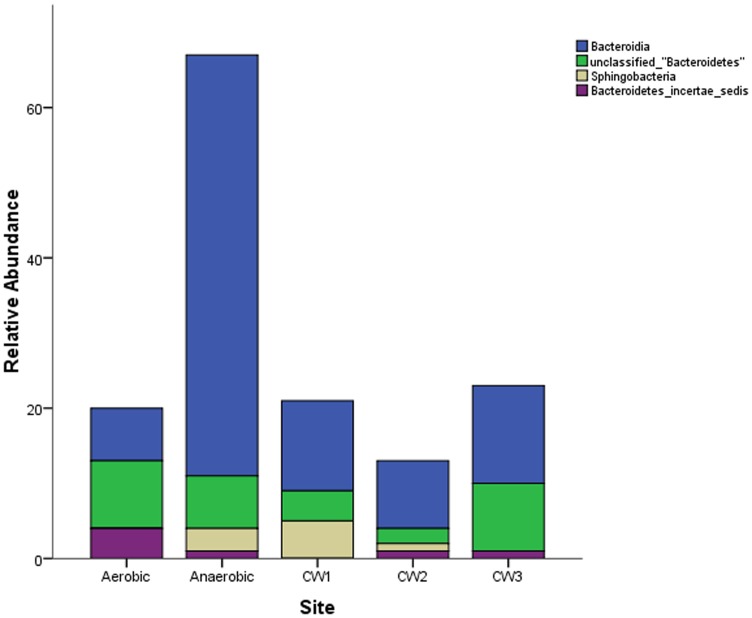
Class level distribution of sequences affiliated to the phylum Bacteroidetes based on the 16S rRNA gene clone libraries from anaerobic- aerobic reactors and three constructed wetland sites.

Considering the class- level abundance of the Phylum Bacteroidetes, members of the class Bacteroidea were the second most abundant in the anaerobic reactor, accounting for 23% of the sequences (Fig. 6). The classes Sphingobacteria, Bacteroidetes incertae sedis and the unclassified Bacteroidetes accounted for less than or equal to 5% of the total sequences in each sample site.

### Evaluation of diversity, coverage and richness

**Figure 7:**
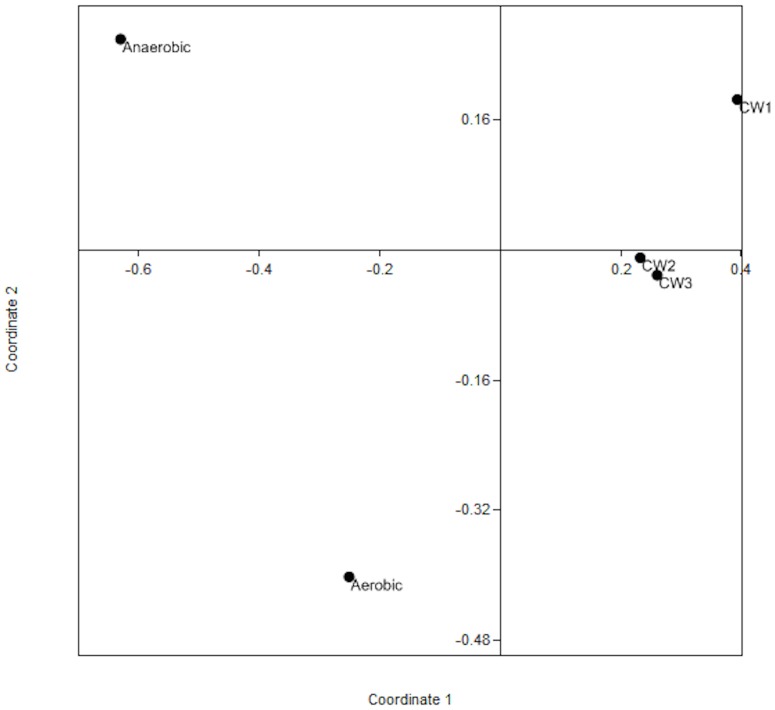
Non- metric multidimensional scaling plot based on Bray- Curtis distance measure for Bacterial Phyla with respect to the different sample sites.

The Shannon-Wiener (H) and Simpson's (D) diversity indices as well as S_ACE_ and Chao I richness estimators indicated that the constructed wetland samples showed the highest diversity and richness, followed by the anaerobic reactor. The aerobic reactor was found to have the least diverse bacterial communities of all the sites in this study (Table 3). Analysis using non-metric Multi-Dimensional Scaling (nMDS) elucidated the rank and position of each sampling site with respect to the bacterial phylotype and frequency (Fig. 7). Unifrac analysis of the sequences from each site further showed significant differences in composition of bacterial communities among aerobic, anaerobic and CW1 of the constructed wetland site while CW2 and CW3 did not show significant differences in their community composition (p>0.01) (Table 4).

**Table 3:** Estimated sample coverage, community richness and diversity estimators of the 16S rRNA gene clone libraries of Modjo Tannery effluent treatment plant samples.

Library (Site)	N (NS)	S	ESC	S_ACE_ *	Chao I*	Shannon (H)	Simpson's (D)
**Anaerobic**	223 (247)	83	0.78	212 (168–278)	170 (123–270)	4.2	0.034
**Aerobic**	151 (184)	58	0.78	141 (109–193)	124 (84–224)	3.9	0.032
**CW1**	125 (162)	84	0.48	524 (397–702)	257 (168–440)	5.0	0.011
**CW2**	131 (180)	97	0.42	325 (217–529)	301 (201–496)	5.3	0.006
**CW3**	131 (204)	86	0.50	425 (313–594)	265 (173–452)	4.9	0.014

Abbreviations: N, Number of clones in each library; NS, Number of unique sequences for each library; S, richness expressed by number of observed OTUs; ESC, estimated sample coverage.

* Values in Parenthesis are 95% confidence intervals.

**Table 4:** Unifrac analysis showing statistical significance (P- values) of differences among the bacterial communities of sites of the biological treatment calculated based on partial sequences of 16S rRNA gene.

	Aerobic	Anaerobic	CW1	CW2	CW3
Aerobic	-	<0.001	<0.001	<0.001	<0.001
Anaerobic		-	<0.001	<0.001	<0.001
CW1			-	0.003	0.001
CW2				-	0.041
CW3					-

P-values of Weighted UniFrac test are calculated based on 1000 permutations (pairwise differences).

Bold values indicate populations not significantly different from each other (P>0.01).

**Figure 8:**
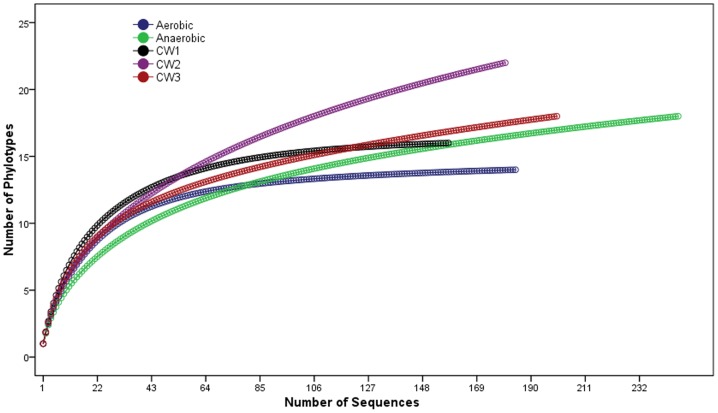
Rarefaction curves of observed bacterial phylotypes for the 16S rRNA clone libraries from anaerobic and aerobic reactors and the three constructed wetland sites.

Rarefaction curve and coverage analysis of the five sites showed curvilinear plots with different saturation levels and coverage indices (Fig. 8 and Table 3). Saturation was reached for the samples from the anaerobic and aerobic reactors indicating the number of sequenced clones was sufficient to identify most bacteria while additional sequencing could be desirable from the constructed wetland (Fig. 8).

**Figure 9:**
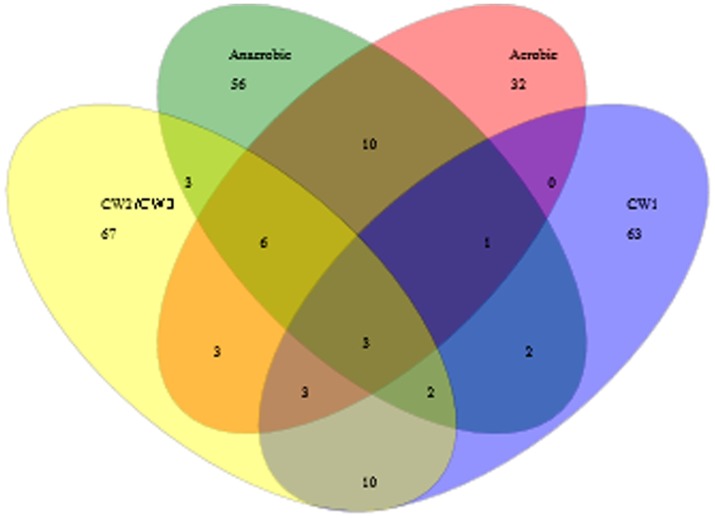
OTUs shared between the aerobic, anaerobic, CW1 and normalized CW2 + CW3 sample sites of the treatment facility as determined by sequence diversity analysis.

To determine the proportions of the bacterial populations that are shared among the aerobic, anaerobic and constructed wetland sites, comparison of the sequences clustered into OTUs from each library was performed. Three OTUs represented by 80 sequences were shared among all the sites (Fig. 9). Taxonomic identification based on BLAST and RDP analysis with a cut- off value of 97% similarity revealed that out of the 80 shared sequences, 26 sequences were members of the Family Clostridiales Incertae Sedis XIII, 36 sequences were members of the phylum Bacteroidetes and 18 sequences belonged to unclassified bacterial group. Based on the finding (Table 4) that clone libraries of CW2 and CW3 of the constructed wetland sites did not show significant variations in their microbial composition, sequences of CW2 and CW3 were pooled and normalized (n = 131) to be represented as a single site in the venn- diagram (Fig. 9).

## Discussion

In this study, the wastewater entering into the treatment system contained high concentrations of organic and inorganic pollutants shown by high levels of COD and BOD. The high concentrations of COD and BOD are in line with a study previously performed on Modjo Tannery effluent by Seyoum Leta et al. [6]. The concentrations of ammonia, total nitrogen, total Cr and sulfide recorded in this study were lower; while salinity and sulfate was higher than the study conducted by Seyoum Leta et al, [6]. Removal of ammonia nitrogen, COD, BOD, TSS, VSS and total chromium by the constructed wetland system was the highest of all the systems indicating the efficiency of the wetland as a secondary treatment system. This is in agreement with a study by Calheiros et al. [36], which indicated the efficiency of constructed wetlands in polishing hypersaline water coming out from a conventional tannery wastewater treatment plant.

Based on the sequence analysis recovered from the clone libraries, the phyla Firmicutes, Proteobacteria and Bacteroidetes represented around 85% of the sequences generated, with the phylum Firmicutes comprising 40% of the total. Calheiros et al. [35], [36] studied the microbial community dynamics in constructed wetlands vegetated with different plants for treating tannery wastewater and reported that Firmicutes dominated the system. Various members of the phylum Firmicutes were also identified in methanogenic oilfields [46], [47]. Members of this phylum are spore- forming bacteria that ensure survival in stressful environmental conditions such as hypersalinity, pH and high oxygen demand, which are typical characteristics of a tannery effluent [6], [34], [36].

A closer look into the dominant phylum Firmicutes indicated that the Class Clostridia was the most dominant bacterial class across all the sample sites, comprising 33% of the total community. Members of this class are strictly anaerobic and they are usually present in anaerobic sewage sludge and Up-flow Anaerobic Sludge Blanket (UASB) reactors [42]. A study involving tannery wastewater treatment in submerged anaerobic membrane bioreactor found the dominance of this Class [43]. Members of Clostridia have been reported from constructed wetlands treating tannery effluents [36]. Other reports indicated the presence of these groups in constructed wetlands treating dairy wash water, which has less pollution load than tannery wastewater [44]. Some of the identified Clostridia genera in this study such as *Proteiniclasticum, Tissierella, Eubacterium* and *Acidaminobacter* have been implicated in the anaerobic degradation of aromatic hydrocarbons like tetrachloroethylene [45]. The unusual dominance of Clostridia in the aerobic reactor in the current study might be due to inadequate oxygen in the particular reactor, suggesting a need for the optimization of aeration in the system.

Betaproteobacteria, the second most abundant bacterial class in the aerobic reactor and wetland sites (10%), comprised the genera *Azospira*, *Thauera* and *Hydrogenophaga* spp., which have been detected in municipal wastewater treatment sites, biogas reactors and contaminated aquifers degrading aromatic compounds [48], [49]. It is possible to infer that members of the class Betaproteobacteria observed in this study may play important roles in the degradation of aliphatic and aromatic retanning compounds in the effluent.

Bacteroidetes, the third abundant phylum in the sampled sites are well known for degrading complex carbon compounds [50]. Previous studies [51] reported the dominance of Bacteroidetes during dye wastewater treatments. Investigations on the microbial communities of a biogas reactor and activated sludge containing chlorinated phenols also reported Bacteroidetes as major members of their communities [52], [53]. A previous study on a bench- scale sequencing batch reactor (SBR) using seed sludge from Modjo Tannery wastewater revealed the significant role played by Bacteroidetes in the degradation of selected retanning chemicals [39]. Therefore, this phylum might be strongly implicated in the degradation of aromatic compounds that are used in the post- tanning process.

Reduced sulfur is one of the characteristics of tannery effluent generated from the use of sodium sulfide, sodium hydrosulfide and from the breakdown of hair during the liming and dehairing process, which contribute to corrosion and malodor [54]. The presence and abundance of members of Deltaproteobacteria, which are exclusively sulfate and sulfur reducing bacteria, is less valued because they may play a role in the release of large concentration of reduced sulfur generated during the production process. In contrast to this scenario, identified members of the genera *Beggiatoa* and *Sulfurimonas* from the class Gammaproteobacteria and Epsilonproteobacteria, respectively, are metabolically well known as sulfur oxidizers [55]. Members of these genera may be implicated in the oxidation of the sulfides generated during the production process and the microbial reduction of sulfur, thus contributing to the high removal efficiency (91%) of sulfides in the treatment system.

Unlike previous reports, Alphaproteobacteria comprised only 2% of sequences in our study. Alphaproteobacteria has been identified as one of the dominant bacterial groups in chromium and arsenic contaminated soils from the vicinity of tanning industries and their effluents [34], [56]. Environmental factors such as salinity have been shown to exert selective pressures on the microbial community [57], [58]. In this study, salinity of the wastewater (9–12 g/l) was relatively higher than the study by Lefebvre et al [34]. Therefore, the high salinity might have accounted for the low abundance of members of the class Alphaproteobacteria.

The anaerobic, aerobic and constructed wetland sites can be considered as distinct habitats in which variations of abiotic factors (such as oxygen availability, wastewater concentration, confinement, surface area), play an important role. The observed high diversity of the microbial community in the constructed wetland sites might be due to the presence of the wetland plant *Phragmites australis* (Cav.), which provides an open system with more surface area for the bacteria. A comparative study by Collins et al [59] conducted on different constructed mesocosm wetland systems, for the remediation of acidic, metal contaminated water from coal pile, indicated that the presence or absence of plants affect the bacterial assemblages in a wetland system, eventually affecting water quality. Within the constructed wetland, the three sample sites did not show wide variability in terms of species diversity and composition, suggesting that bacteria colonized the root zones in a similar fashion despite the observed variation in the vegetation density across the three sites.

The performance of a biological wastewater treatment system has long been evaluated by operational parameters such as biochemical oxygen demand and chemical oxygen demand, which are usually considered to account for the stability of a treatment system [60]. It is worthwhile to consider the microorganisms responsible for the clarification process as one of the factors affecting treatment plants' performance and stability [23]. In the current study, the overall high bacterial diversity in the anaerobic and aerobic reactors as well as the wetland sites might contribute to the performance of the system, which was expressed in the removal efficiencies of the integrated system with regard to the major pollutants studied.

The findings of this study provides a snapshot of the composition and structure of bacterial community in the integrated anaerobic- aerobic biological reactors connected with a constructed wetland for the treatment of a complex tannery wastewater. Several microbial groups have been identified with putative critical roles in the removal of carbon, nitrogen and sulfur containing compounds from the wastewater. In order to have conclusive information on the bacterial population dynamics playing key roles in removal of these pollutants, it is important to perform a longitudinal investigation of microbes in each component of the treatment system as part of a routine measurement of biotic and abiotic factors over time.
