# Correction: Microbial Community Structure and Diversity in an Integrated System of Anaerobic-Aerobic Reactors and a Constructed Wetland for the Treatment of Tannery Wastewater in Modjo, Ethiopia

**DOI:** 10.1371/journal.pone.0128053

**Published:** 2015-05-04

**Authors:** Adey Feleke Desta, Fassil Assefa, Seyoum Leta, Francesca Stomeo, Mark Wamalwa, Moses Njahira, Appolinaire Djikeng

The seventh author’s name is incorrect. The correct name is Appolinaire Djikeng.
